# Genomic analysis of Lassa virus from the 2018 surge in Nigeria

**DOI:** 10.1056/NEJMoa1804498

**Published:** 2018-10-17

**Authors:** Katherine J. Siddle, Philomena Eromon, Kayla G. Barnes, Judith U. Oguzie, Samar Mehta, Ikponmwonsa Odia, Rickey Shah, Patrick Brehio, Sarah M. Winnicki, Christopher Iruolagbe, John Aiyepada, Eghosa Uyigue, Patience Akhilomen, Grace Okonofua, Bridget Chak, Dylan Kotliar, Blessing Osiemi, Ekene Muoebonam, Michael Airende, Rachael Ukpetina, Iguosadolo Nosamiefan, Paul Oluniyi, Ephraim Ogbaini-Emovon, Mahan Nekouin, Onikepe A. Folarin, Stephen F. Schaffner, Robert F. Garry, Kristian G. Andersen, Daniel J. Park, Nathan L. Yozwiak, Bronwyn L. MacInnis, George Akpede, Sylvanus Okogbenin, Peter Okokhere, Pardis C. Sabeti, Christian T. Happi

**Affiliations:** Broad Institute of MIT and Harvard, Cambridge, Massachusetts, USA; Center for Systems Biology, Department of Organismic and Evolutionary Biology, Harvard University, Cambridge, Massachusetts, USA.; African Center of Excellence for Genomics of Infectious Disease (ACEGID), Redeemer's University, Ede, Osun State, Nigeria.; Broad Institute of MIT and Harvard, Cambridge, Massachusetts, USA; Center for Systems Biology, Department of Organismic and Evolutionary Biology, Harvard University, Cambridge, Massachusetts, USA; Department of Immunology and Infectious Diseases, Harvard T.H. Chan School of Public Health, Harvard University, Boston, Massachusetts, USA.; African Center of Excellence for Genomics of Infectious Disease (ACEGID), Redeemer's University, Ede, Osun State, Nigeria; Department of Biological Sciences, College of Natural Sciences, Redeemer’s University, Ede, Osun State, Nigeria.; Broad Institute of MIT and Harvard, Cambridge, Massachusetts, USA; Beth Israel Deaconess Medical Center, Division of Infectious Diseases, Boston, Massachusetts, USA.; Institute of Lassa Fever Research and Control, Irrua Specialist Teaching Hospital, Irrua, Edo State, Nigeria.; Center for Systems Biology, Department of Organismic and Evolutionary Biology, Harvard University, Cambridge, Massachusetts, USA.; Broad Institute of MIT and Harvard, Cambridge, Massachusetts, USA.; Broad Institute of MIT and Harvard, Cambridge, Massachusetts, USA.; Institute of Lassa Fever Research and Control, Irrua Specialist Teaching Hospital, Irrua, Edo State, Nigeria.; Institute of Lassa Fever Research and Control, Irrua Specialist Teaching Hospital, Irrua, Edo State, Nigeria.; Department of Biological Sciences, College of Natural Sciences, Redeemer’s University, Ede, Osun State, Nigeria; Institute of Lassa Fever Research and Control, Irrua Specialist Teaching Hospital, Irrua, Edo State, Nigeria.; Institute of Lassa Fever Research and Control, Irrua Specialist Teaching Hospital, Irrua, Edo State, Nigeria.; Institute of Lassa Fever Research and Control, Irrua Specialist Teaching Hospital, Irrua, Edo State, Nigeria.; Broad Institute of MIT and Harvard, Cambridge, Massachusetts, USA; Center for Systems Biology, Department of Organismic and Evolutionary Biology, Harvard University, Cambridge, Massachusetts, USA.; Broad Institute of MIT and Harvard, Cambridge, Massachusetts, USA; Center for Systems Biology, Department of Organismic and Evolutionary Biology, Harvard University, Cambridge, Massachusetts, USA.; Institute of Lassa Fever Research and Control, Irrua Specialist Teaching Hospital, Irrua, Edo State, Nigeria.; Institute of Lassa Fever Research and Control, Irrua Specialist Teaching Hospital, Irrua, Edo State, Nigeria.; Institute of Lassa Fever Research and Control, Irrua Specialist Teaching Hospital, Irrua, Edo State, Nigeria.; Institute of Lassa Fever Research and Control, Irrua Specialist Teaching Hospital, Irrua, Edo State, Nigeria.; African Center of Excellence for Genomics of Infectious Disease (ACEGID), Redeemer's University, Ede, Osun State, Nigeria.; African Center of Excellence for Genomics of Infectious Disease (ACEGID), Redeemer's University, Ede, Osun State, Nigeria; Department of Biological Sciences, College of Natural Sciences, Redeemer’s University, Ede, Osun State, Nigeria.; Institute of Lassa Fever Research and Control, Irrua Specialist Teaching Hospital, Irrua, Edo State, Nigeria.; Faculty of Arts and Sciences, Harvard University, Cambridge, Massachusetts, USA.; African Center of Excellence for Genomics of Infectious Disease (ACEGID), Redeemer's University, Ede, Osun State, Nigeria; Department of Biological Sciences, College of Natural Sciences, Redeemer’s University, Ede, Osun State, Nigeria.; Broad Institute of MIT and Harvard, Cambridge, Massachusetts, USA; Center for Systems Biology, Department of Organismic and Evolutionary Biology, Harvard University, Cambridge, Massachusetts, USA; Department of Immunology and Infectious Diseases, Harvard T.H. Chan School of Public Health, Harvard University, Boston, Massachusetts, USA.; Tulane Health Sciences Center, Tulane University, New Orleans, LA 70118, USA.; Department of Immunology and Microbial Science, The Scripps Research Institute, La Jolla, California, USA; Scripps Translational Science Institute, La Jolla, California, USA; Department of Integrative Structural and Computational Biology, The Scripps Research Institute, La Jolla, California, USA.; Broad Institute of MIT and Harvard, Cambridge, Massachusetts, USA.; Broad Institute of MIT and Harvard, Cambridge, Massachusetts, USA; Center for Systems Biology, Department of Organismic and Evolutionary Biology, Harvard University, Cambridge, Massachusetts, USA.; Broad Institute of MIT and Harvard, Cambridge, Massachusetts, USA; Department of Immunology and Infectious Diseases, Harvard T.H. Chan School of Public Health, Harvard University, Boston, Massachusetts, USA.; Institute of Lassa Fever Research and Control, Irrua Specialist Teaching Hospital, Irrua, Edo State, Nigeria.; Institute of Lassa Fever Research and Control, Irrua Specialist Teaching Hospital, Irrua, Edo State, Nigeria.; Institute of Lassa Fever Research and Control, Irrua Specialist Teaching Hospital, Irrua, Edo State, Nigeria; Department of Medicine, Irrua Specialist Teaching Hospital, Irrua, Nigeria; Department of Medicine, Faculty of Clinical Sciences, Ambrose Alli University, Ekpoma, Nigeria.; Broad Institute of MIT and Harvard, Cambridge, Massachusetts, USA; Center for Systems Biology, Department of Organismic and Evolutionary Biology, Harvard University, Cambridge, Massachusetts, USA; Department of Immunology and Infectious Diseases, Harvard T.H. Chan School of Public Health, Harvard University, Boston, Massachusetts, USA; Howard Hughes Medical Institute, Chevy Chase, Maryland, USA.; African Center of Excellence for Genomics of Infectious Disease (ACEGID), Redeemer's University, Ede, Osun State, Nigeria; Department of Biological Sciences, College of Natural Sciences, Redeemer’s University, Ede, Osun State, Nigeri; Institute of Lassa Fever Research and Control, Irrua Specialist Teaching Hospital, Irrua, Edo State, Nigeria.

## Abstract

In early 2018 Nigeria experienced an unprecedented increase in Lassa fever cases with widespread geographic distribution. We report 77 Lassa virus genomes generated from patient samples, 14 from 2018, to investigate whether recent changes in the virus genome contributed to this surge. Our data argue that the surge is not attributable to a single Lassa virus variant, nor has it been sustained by human-to-human transmission. We observe extensive viral diversity structured by geography, with major rivers appearing to act as barriers to migration of the rodent reservoir. Together our results support that the 2018 Lassa fever surge was driven by crossspecies transmission from local rodent populations of multiple viral variants from different lineages.

## INTRODUCTION

Lassa fever is a viral hemorrhagic disease endemic to parts of Western Africa that causes over 300,000 cases and 3,000 fatalities per year^1^. It has been recognized by the World Health Organization (WHO) and the Coalition for Epidemic Preparedness Innovations (CEPI) as a significant threat to global health and in need of urgent R&D attention^2-4^. Despite the burden of Lassa virus, there is currently no approved vaccine, and the only available pharmacologic therapy is early intravenous administration of the antiviral ribavirin^5-7^. In early 2018 there was a marked increase in Lassa fever cases in Nigeria: by early March, Nigeria had more confirmed cases (394) than in any previous year. Confirmed cases were observed in 19 Nigerian states, with an estimated case fatality rate of approximately 25%^8^. The factors underlying this increase were not known, raising concern among public health officials that something had fundamentally changed about this endemic disease. 

In a presumed Lassa fever outbreak, genomic analysis of contemporaneous Lassa virus in samples from infected patients can complement conventional epidemiological data by determining whether changes to intrinsic properties of the virus explain the increase in cases. In particular, viral genomic analysis can rapidly assess whether a novel variant or specific viral lineage, or a change in viral transmission route is associated with the case surge. Most human Lassa virus infections result from contact with infected *Mastomys natalensis* (the major natural reservoir^9^) or their excreta, but human-to-human transmission has been documented in hospital settings and is a focus of public health monitoring^10,11^. Previous retrospective investigation of the genomic epidemiology of Lassa virus in Nigeria between 2008 and 2014 showed extensive genetic diversity across the region and provided support for predominantly reservoir-to-human transmission^12^. Subsequent studies have extended the known genetic diversity of Lassa virus, of which there are at least four firmly established lineages^13^, as well as its geographic range in Western Africa^14,15^. Against this backdrop, genomic analysis of Lassa virus during the 2018 can quickly establish changes in the viral genome associated with period of increased Lassa fever cases. 

Here we report near real-time genome analysis of Lassa virus from patients from January to March 2018, undertaken at the African Center of Excellence for Genomics of Infectious Disease (ACEGID), at Redeemer’s University in Nigeria. These data provide important genomic context to the recent Lassa fever surge and further resolve the geographic structure of the endemic Lassa virus population across Nigeria. 

## METHODS

### Patient sample collection

We obtained patient samples through a study evaluated and approved by Institutional Review Boards (IRBs) at Irrua Specialist Teaching Hospital (ISTH, Irrua, Nigeria), Redeemer’s University (Ede, Osun State, Nigeria), and Harvard University (Cambridge, Massachusetts). Study staff obtained informed consent from participants enrolled in the research study at ISTH. In addition, some samples were included under a waiver of consent to facilitate rapid public health response as the research involved minimal risk to the subjects. Samples from suspected Lassa fever cases were tested for Lassa virus by RT-qPCR (reverse transcriptase - quantitative polymerase chain reaction) at the clinical diagnostics laboratory at ISTH. We de-identified samples and obtained demographic and clinical data in line with ethical approvals. We prepared a subset of samples with positive Lassa virus RT-qPCR diagnosis, spanning the time frame of the surge, for sequencing. 

### Viral sequencing

We extracted RNA from patient plasma using the QiAmp viral RNA mini kit (Qiagen) or Pathogen RNA/DNA kit (MagMax) according to the manufacturer’s instructions. We removed contaminating DNA by DNase treatment, synthesized cDNA, and prepared sequencing libraries using the Nextera XT kit (Illumina) as previously described^16^. We constructed sequencing libraries directly from clinical samples without culture or other intervention. We extracted, prepared, and sequenced samples from 2018 at ACEGID, Redeemer’s University, Ede, Osun State, Nigeria, and those from prior to 2018 at ACEGID or the Broad Institute, Cambridge, MA, USA. We additionally performed replicate sequencing of samples from 2018 at the Broad Institute for intra-host variant detection. We sequenced all samples using Illumina MiSeq and HiSeq 2500 machines with 100 nucleotide paired-end reads. 

### Genomic data analysis

We analyzed sequencing data using our publicly available software viral-ngs v1.19.2^17,18^ implemented on the DNAnexus cloud-based platform. Briefly, we demultiplexed individual libraries, removed reads mapping to the human genome and to other known technical contaminants (e.g. sequencing adapters), and filtered the remaining reads against previously published Lassa virus genomes. We performed *de novo* assembly using Trinity^19^ and scaffolded contigs against one of three Lassa virus reference genomes (KM821997-8, GU481072-3, KM821772-3), representing the major viral lineages (II, III and IV). We used Kraken v0.10.6^20^ in viral-ngs to identify other viral taxa present in the samples. To do so, we first built a database that encompassed the known diversity of all viruses that infect humans (similar to that described elsewhere^21^, but without insect species). We searched for viral species detected in the samples with a read count at least 1.5x greater than that of any viral taxon identified in negative control samples and manually investigated any potential hits. We detected intra-host variants in samples from 2018 using V-Phaser 2^22^ implemented in viral-ngs v1.19.2 using default parameters. To do so, we leveraged data from independently prepared replicate sequencing libraries for 13 of the 14 samples. 

In order to construct the phylogenetic tree of Lassa virus, we performed a multiple sequence alignment of our new genomes with a set of 193 previously published Lassa virus genomes from Nigeria, Sierra Leone, Liberia, and Côte d’Ivoire^12^. We performed codon-based multiple sequence alignments of the NP and GPC sequences using MAFFT^23^. We estimated maximum likelihood phylogenies of concatenated alignments of NP and GPC using IQ-TREE v1.5.5^24,25^ using a GTR substitution model and ultrafast bootstrapping. To create time-aware phylogenies for the Nigerian lineage II sequences, we then performed Bayesian phylogenetic analyses using the program BEAST v1.8.4^26^, incorporating the collection date for each sequence. We included GPC and NP lineage II alignments as separate partitions. We used a model consisting of an SRD06 codon-aware nucleotide substitution model^27^, an uncorrelated relaxed clock with a lognormal distribution, and a Bayesian SkyGrid coalescent tree prior. All of the Bayesian analyses were run for 200 million MCMC steps, sampling parameters and trees every 5,000 generations. Maximum-clade credibility trees summarizing all MCMC samples were generated using TreeAnnotator v1.8.4 with a burn-in rate of 10%. 

## RESULTS

### Lassa fever case burden at ISTH in 2018

**Figure 1: Incidence of Lassa virus in Nigeria in recent years.**
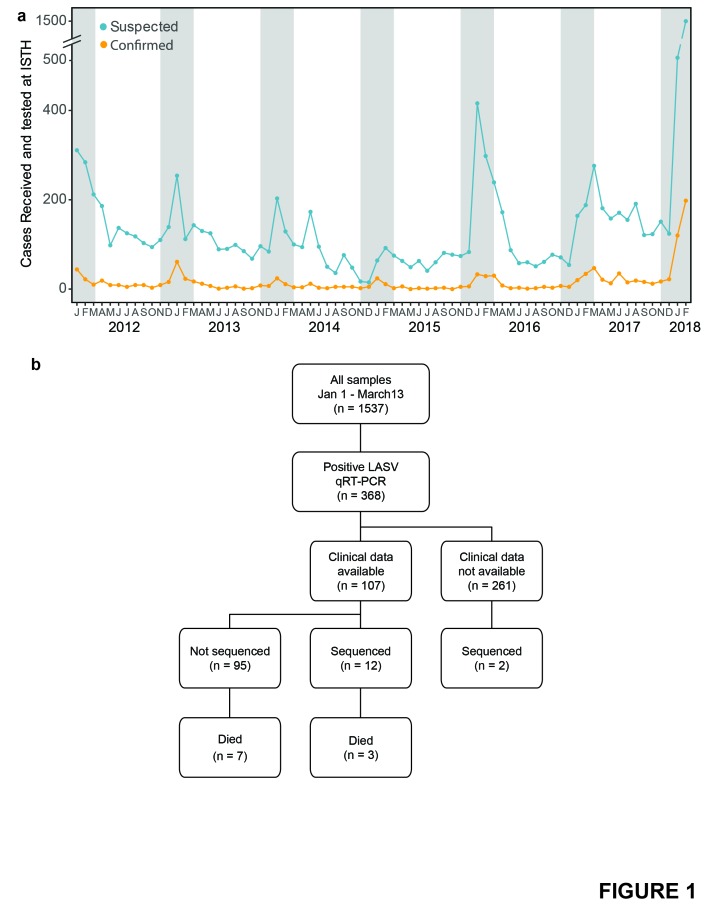
a) Number of clinically suspected Lassa fever cases (blue) and RT-qPCR-positive cases (orange) tested at ISTH monthly from January 2012 to February 2018. Counts are those reported by ISTH. Gray shading denotes dry season months in Nigeria, when Lassa cases are typically highest. b) Samples processed at ISTH from January 1 to March 13, 2018. Outcome data, where available, are up to date as of March 22.

The ISTH Lassa ward, with 16 beds, is the largest Lassa fever facility in Nigeria and a major diagnostic referral center, receiving suspected Lassa fever patient samples from across the country. From January 1 to March 13, 2018, ISTH tested over 1500 clinically suspected Lassa fever cases, of which 368 were RT-qPCR-positive for Lassa virus (Fig. 1A & 1B). This number, which represents the majority of confirmed cases in Nigeria during this period, is markedly higher than that observed in previous years (Fig. 1A). There is a wide distribution of ages (Fig. S1A) and geographic source of confirmed cases (Fig. S1B), as previously observed for Lassa fever^28^. We did observe an approximate 2:1 male-to-female ratio among confirmed cases, in contrast to previous conclusions that Lassa fever does not exhibit sex disparity^11^, though it would be difficult to determine whether this reflects a true difference, given the sampling bias inherent in clinical surveillance. Patients included healthcare workers, farmers, lawyers and students, demonstrating the broad reach of the 2018 surge. 

### Lassa virus sequencing of patient samples from 2018 surge

To investigate the viral population underpinning this surge, we performed unbiased sequencing and assembled Lassa virus genomes on a subset of RT-qPCR-positive patient samples (Fig. 1B). We obtained complete or high-quality partial Lassa virus genomes from 14 out of 26 RTqPCR- positive patient samples. Table S1 summarizes sequence and assembly quality metrics for these samples. The mean unambiguous assembly length of these genomes was 9,039 bases (4,450-10,610) and mean coverage depth was 193x (1-1,834). 12 samples did not readily produce high-quality Lassa virus genomes. We did not find evidence consistent with other pathogenic viral infections in any of the samples from 2018, with the depth of sequencing available. 

The 14 patients from whom we assembled Lassa virus genomes were reflective of the demographic characteristics of the larger cohort, including age (Fig. S1A), sex (Table 1) and geographic distribution (Fig. S1B). Clinically, the picture is of a nonspecific febrile illness that sometimes develops into a bleeding diathesis. Hemorrhage was documented in 2 of the 3 patients who died and in at least 3 of the 9 who recovered, suggesting a range of disease severity^29^. This is broadly consistent with clinical descriptions of Lassa fever: patients typically present with nonspecific symptoms, including fever, headache, malaise and general weakness, often indistinguishable from malaria or common viral diseases. Case fatality rates, though challenging to determine, are estimated at 15-20% among hospitalized cases11, though a recent study estimated case fatality rates in Nigeria during 2015-2016 to be 60%^30^. 

**Table 1:** Demographic data and symptoms as reported for 14 patients whose virus was sequenced at ACEGID in 2018.

ID	Age/Sex	State	Symptom onset	Sample Collection	Symptoms	Outcome	Genbank #
0026	32y M	Edo	2017-12-29	2018-01-07	Fever, headache, weakness	Recovered	MH157043, MH157046
0097	44y M	Ondo	2018-01-08	2018-01-15	Fever, abdominal pain, sore throat, weakness	Recovered	MH157049, MH157035
0541	18y F	Edo	2018-01-30	2018-02-01	Fever, headache, abdominal pain	Recovered	MH157048, MH157044
0611	41y F	Ebonyi	2018-02-02		Fever, headache, unspecified bleeding		MH157039
0664	20y F	Ondo	2018-02-04		Fever, abdominal pain		MH157053, MH157028
0959	32y M	Edo	2018-02-03	2018-02-12	Fever, vomiting, diarrhea, haematuria, weakness	Died	MH157042, MH157032
0998	32y M	Edo	2018-02-05	2018-02-13	Fever, abdominal pain, sore throat, cough, weakness	Recovered	MH157030
1024	25y M	Edo	2018-02-01	2018-02-14	Fever, headache, cough, general body pain, weakness	Recovered	MH157047, MH157037
1079	43y M	Ondo	2018-02-07	2018-02-15	Fever, headache, abdominal pain, vomiting, diarrhea, bleeding, sore throat, weakness	Recovered	MH157029, MH157038
1177	33y M	Edo	2018-02-04	2018-02-18	Fever, weakness, abdominal pain, sore throat, haematemesis	Died	MH157036, MH157034
1375	48y M	Ondo	2018-02-16	2018-02-23	Fever, abdominal pain, headache, sore throat, vomiting, diarrhea, weakness	Died	MH157033, MH157045
1381	30y F	Kogi	2018-02-08	2018-02-23	Fever, abdominal pain, headache, sore throat, diarrhea, haematemesis	Recovered	MH157040, MH157041
1392	14y F	Edo	2018-02-16	2018-02-24	Fever, vomiting, cough, haematuria	Recovered	MH157051, MH157052
1643	27y M	Edo	2018-02-25	2018-03-05	Fever, headache, sore throat	Recovered	MH157031, MH157050

**Figure 2. Distribution of Lassa virus genetic diversity in Nigeria.**
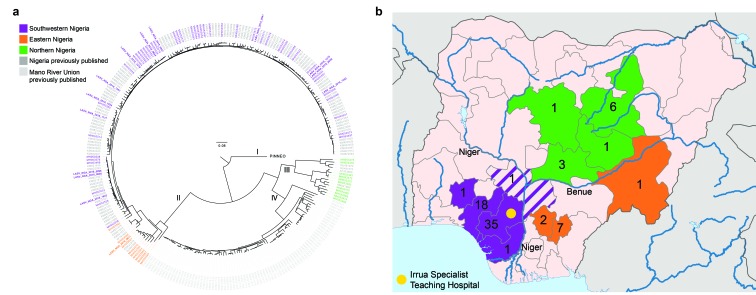
a) Maximum likelihood phylogenetic tree of the S segment of the Lassa virus genome. The tree incorporates the 77 new sequences presented here alongside 193 previously published sequences from Nigeria and the Mano River Union (in gray). The 77 new samples are coloured by geographic region in which the patient resides. Samples from 2018 are in bold. b) Map of Nigeria highlighting the states from which the 77 new sequences originate and the number of samples from each state. Colours are the same as in A. Kogi state, at the intersection of the 2 rivers, is shown in striped purple reflecting the clustering of the single sequenced sample from this state with others from the southwest region in A. The location of Irrua Specialist Teaching Hospital is marked in yellow.

To look for evidence of a novel viral genetic variant or sustained human-to-human transmission driving the 2018 case surge, we performed phylogenetic analysis of these 14 genomes from 2018. A maximum likelihood phylogeny shows that the 2018 genomes fall within previously known Lassa virus diversity in Nigeria (Fig. 2A) and do not display substantial clustering by date of sampling, consistent with multiple zoonotic transmissions. Estimated dates for the branch points of closely related 2018 samples in this small dataset, which are in the range of years, do not support a surge in human-to-human transmission in 2018 (Fig. S2). We also identified several intra-host Single Nucleotide Variants at a minor allele frequency >5% in 5 of the 14 patient samples, indicating some virus evolution and *de novo* mutation within hosts. However, none of these variants were in coding regions and only 1 was shared between samples (Table S2). 

### Genomic epidemiology of Lassa virus in Nigeria

We next assessed these genomes in the context of the recent history of Lassa virus diversity in Nigeria, to determine whether the larger picture showed patterns that could help explain the recent surge. To do so, we extended our dataset to include 63 new Lassa virus genomes from RT-qPCR-positive patient samples collected at ISTH between August 2015 and November 2016 (BioProject accession PRJNA436552; Table S3). The patients resided in 11 states, with most (68%) coming from Edo and Ondo. This combined dataset considerably expands and updates previous phylogenetic trees of Lassa virus in Nigeria. 

Samples from 2015-2018 cluster geographically on the phylogenetic tree. All eleven samples sequenced here from northern Nigeria fall into lineage III (Fig. 2B), increasing our sampling of this lineage more than threefold. These samples confirm the high genetic diversity of this lineage and make clear that it is a regionally defined variant of Lassa virus. Our dataset further identifies a separation in lineage II between samples from southwestern and eastern states, with samples from the eastern states of Ebonyi, Taraba and Anambra forming a distinct sublineage (Fig. 2B). This pattern of distinct regional lineages, each internally diverse, indicates that Lassa virus has remained stably separated in the rodent populations of these regions; for example, the most recent common ancestor of lineage II occurred around 235 years ago (95% CI: 187-283; Fig. S2). 

The observed clustering aligns with the courses of the Niger and Benue rivers in Nigeria (Fig. 2B), suggesting that these major rivers present natural barriers to *Mastomys* rodents. This pattern further supports a key role for the rodent reservoir, and not humans, in the ongoing transmission of Lassa virus. Together with the long branch lengths of these groups – suggestive of extensive, uncaptured Lassa virus diversity in these regions – these results indicate sequestering of the rodent population and their associated Lassa virus lineages in these regions. 

## DISCUSSION

We undertook genome sequencing of Lassa virus from patient samples to assess whether intrinsic properties of the viral genomes contributed to the recent increase in Lassa fever cases in Nigeria. In our initial dataset of 14 genomes from 2018, we observe no evidence that either a particular viral variant or extensive human-to-human transmission drove the surge. Lassa virus genomes both from 2018 and from 2015-16 were broadly distributed across different Lassa virus lineages, suggesting that no single variant was associated with the recent increase in Lassa fever. Furthermore, we do not observe phylogenetic clustering of Lassa virus genomes from samples collected close in time, as would be expected if this surge were driven by humanto- human transmission. The absence of these patterns supports the assertion that Lassa virus transmission in 2018 was sustained by multiple distinct cross-species transmission events, consistent with previous observations^12,13^. These findings suggest future studies of the 2018 increase in cases prioritize investigating changes in the rodent reservoir population as well as the role of heightened surveillance and clinical awareness^31^. 

The data reported here also improve our understanding of Lassa virus genetic diversity across Nigeria, revealing clear geographic population structure and extensive diversity in regions that have previously been poorly sampled. Intriguingly, we see substantial genetic divergence between regions demarcated by two major rivers, suggesting the importance of established, local rodent populations in sustaining Lassa virus transmission^13^. Together, these results reaffirm the need for widespread geographic sampling of Lassa virus in Nigeria, including more extensive sampling from the rodent reservoir, in order to better understand its genetic diversity. A comprehensive knowledge of this diversity is critical for development of urgently needed Lassa fever diagnostics and vaccines^2,3^. 

The 2018 Lassa fever cases in this study were sequenced locally in Nigeria, leveraging longterm investments to establish local, responsive genomics laboratory capacity. These data were then rapidly shared with key public health organisations, who recognized the value of genomic data to inform case tracking and management. Continued development of local genomics capacity and growth of these collaborations will facilitate a more agile and integrated approach to outbreaks. We envision a model for genomics-informed outbreak investigation in which locally generated sequence data is rapidly integrated with traditional epidemiological data to refine response strategies. 

## Supplementary Materials

Supplementary Materials
