# Supplementary material for: Genomic analysis of Lassa virus from the 2018 surge in Nigeria
Source: N Engl J Med. Author manuscript; Available in PMC 2018 Oct 18. (PMC6181183; doi:10.1056/NEJMoa1804498)
Supplement: Supplementary Materials [file NEJMoa1804498_Siddle-Supplement.pdf]

Supplementary Materials

1. **Figure S1.** Epidemiology of patients confirmed RT-qPCR-positive for Lassa virus at ISTH.

2. **Figure S2.** Time-aware phylogenetic tree of Lassa virus diversity in lineage II.

3. **Table S1.** Assembly data for 14 patients whose virus was sequenced at ACEGID in 2018.

4. **Table S2.** Intra-host Single Nucleotide Variants present in samples from 2018 at a minor allele frequency above 5%

5. **Table S3.** Assembly data for virus from 63 patients from 2015-2016.

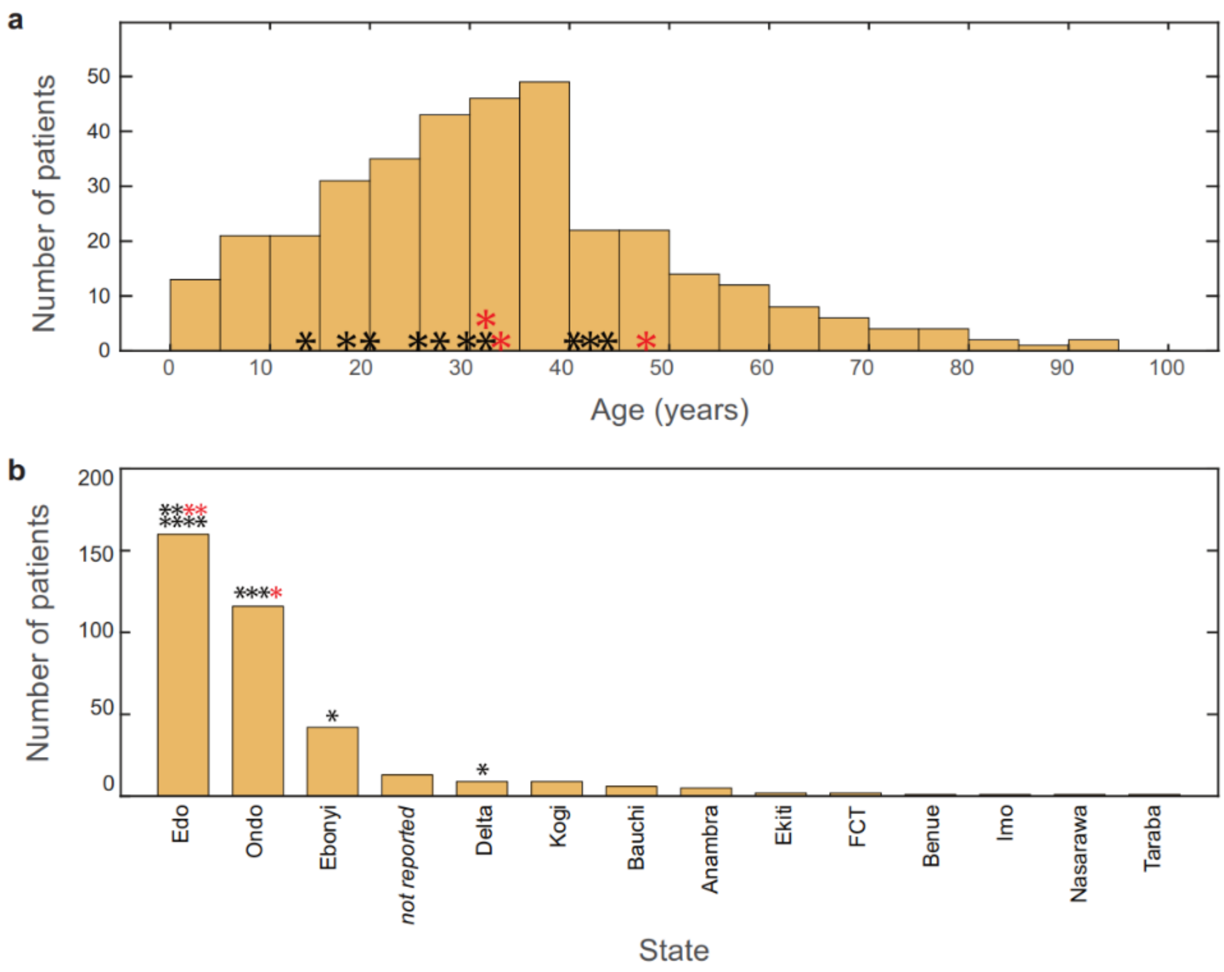

Figure S1. Epidemiology of patients confirmed RT-qPCR-positive for Lassa virus at ISTH. a) Histogram of patient ages. b) Recorded place of origin. For patients seen at ISTH, this refers to the state in which they live, while for samples sent from outside facilities, this refers to the location of the facility that sent the sample. Asterisks show ages of the patients from whom we obtained Lassa virus genomes; red asterisks indicate patients who died.

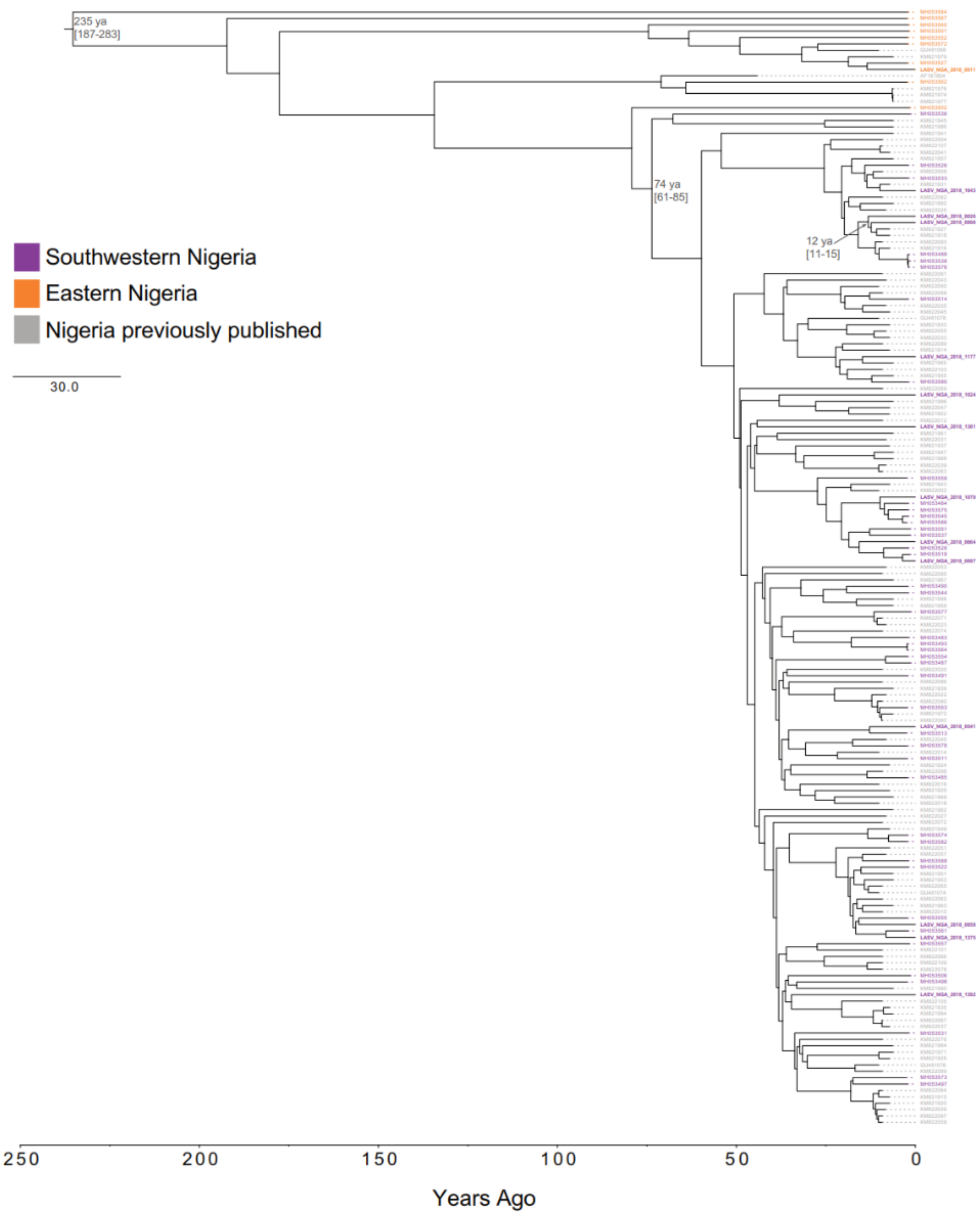

Figure S2. Time-aware phylogenetic tree of Lassa virus diversity in lineage II. Bayesian phylogenetic analysis of the GPC and NP genes of Lassa virus sequences from lineage II, constructed using the program BEAST. Node labels are the mean height with 95% confidence intervals of selected key nodes in the tree.

Table S1. Assembly data for the 14 genomes from 2018 sequenced at ACEGID.

ID	Initial reads	Post-depletion	Post-filtering	Assembly length (bp)	Mean coverage depth
0026	2301852	9322	726	10120	3
0097	8607556	385672	370028	10629	1834
0541	1530402	116668	2742	10367	19
0611	5194502	17894	2826	9627	22
0664	2098036	40924	4334	10469	21
0959	1031416	31042	22398	10528	130
0998	4396476	65000	360	9495	1
1024	1756646	12142	3042	10433	24
1079	2354328	26522	19510	10593	149
1077	1307102	148630	126464	10510	646
1375	4661070	11090	3976	10480	30
1381	3281066	6144	1128	10404	8
1392	4947710	5384	466	9566	2
1643	1678382	5522	2144	10397	16

Table S2. Intra-host Single Nucleotide Variants present in samples from 2018 at a minor allele frequency above 5%.

ID	Reference Genome	Position	Alleles	Frequency	Variant Type	Base Change	Codon Change	Gene
1381	KM821998	646	T,A	0.6097561	synonymous_variant	588T>C,588T>A	Gly196Gly	GPC
0664	KM821998	1570	G,A	0.12903226	intergenic_region	n.1570G>A		GPC-NP
1177	KM821998	1570	G,A	0.05625879	intergenic_region	n.1570G>A		GPC-NP
1177	KM821998	1593	G,A	0.08327025	intergenic_region	n.1593G>A		GPC-NP
1381	KM821998	1692	T,C	0.525	synonymous_variant	1617A>G	Ala539Ala	NP
1381	KM821998	2115	C,T	0.36781609	synonymous_variant	1194G>T,1194G>A	Val398Val	NP
1381	KM821998	2184	G,A	0.39772727	synonymous_variant	1125C>T	Asp375Asp	NP
1381	KM821998	2307	A,G	0.625	synonymous_variant	1002T>C	Thr334Thr	NP
0026	KM821998	2499	A,G	0.26206897	synonymous_variant	810T>A,810T>C	Val270Val	NP
0026	KM821998	2736	A,T	0.84868421	synonymous_variant	573T>A,573T>C	Gly191Gly	NP
1024	KM821998	2898	T,C	0.944	synonymous_variant	411A>G	Arg137Arg	NP
1381	KM821998	3132	C,T	0.8	synonymous_variant	177G>A	Arg59Arg	NP
1024	KM821998	3153	G,A	0.05882353	synonymous_variant	156C>T	Arg52Arg	NP

Table S3. Assembly data for the 63 genomes from 2015-16.

ID	Initial reads	Post-depletion	Post-filtering	Assembly length (bp)	Mean coverage depth
15_0543	28209962	1851312	1526656	10162	5517
15_0547	23500328	21727702	21607192	10201	196
15_0574	20208066	18825816	18770266	10201	181
15_0681	26565728	22980010	22381002	10151	176
15_0682	30190572	736336	616400	10536	2234
15_0687	23407374	14056890	11199076	10225	150
15_0736	33216866	29509366	28842122	10478	208
15_0789	18419446	664076	249652	9917	1337
15_0821	14537986	7441288	6131050	10209	140
15_0831	15519946	6984412	4741840	9997	126
16_0009	6446958	4919972	4507576	10195	101
16_0017	21238678	2468998	2348014	10569	8508
16_0035	9004614	7432084	6260422	10599	111
16_0089	3797110	238248	102664	10453	558
16_0104	15025448	1516612	1464140	10199	6698
16_0133	13811638	265196	116534	10097	694
16_0141	23663394	1032120	509146	10567	1820
16_0146	16771370	1145664	1065528	10564	2744
16_0176	57932384	4020902	2967740	10573	10195
16_0187	15428370	14507704	14444604	10083	172
16_0199	5805912	2967126	1892456	10091	75
16_0314	1010408	147796	82024	10035	467
16_0419	5338738	357318	204404	9894	1207
16_0447	11508866	253272	150886	10567	644
16_0543	30161622	2022716	1767498	10224	6337
16_0565	1796822	257476	166176	10097	780
16_0572	12262188	3475524	3348264	10196	124
16_0610	10116502	8380106	8073802	10174	137
16_0621	38913094	1416614	836496	10056	4066
16_0701	25025396	575396	410572	10521	1236
16_0702	50566192	370810	21050	10610	33
16_0703	19789754	107566	16670	10617	93
16_0717	40534688	1510612	1448474	10454	4244
16_0753	20034200	649254	100600	10639	553
16_0759	89151760	4151528	3978836	10604	7262
16_0777	23316382	1150136	570720	10233	2254
16_0779	17219434	863530	278950	10317	51
16_0917	11321820	21332	14544	10032	70
16_0919	10673954	359216	321056	10610	1461
16_1006	15599566	186740	100956	10627	34
16_1026	25537104	203242	46320	10578	12
16_1069	693442	50504	36634	10621	263
16_1070	2114510	8028	5122	10517	40
16_1123	31804248	219334	34530	10623	140
16_1150	14489942	118524	93440	10649	388
16_1174	1113206	123942	95948	10637	667
16_1207	47125432	242774	114166	10625	428
16_1250	2938210	132496	130234	10640	833
16_1306	668104	14898	2600	10096	19
16_1311	2258500	33672	29908	10629	228
16_1398	3744952	66892	16804	10635	71
16_1423	3266362	605176	593414	10635	3533
16_1462	2656794	27936	7756	10616	56
16_1472	16766356	1564894	1478496	10640	112
16_1474	22675078	101340	31924	10630	105
16_1496	16415340	237092	18048	10582	71
16_1541	15828518	376340	349966	10026	1703
16_1546	9584668	59986	56902	10047	359
16_1568	19594432	60278	44902	10618	126
16_1591	21269020	77528	18418	10597	78
16_1604	13529682	510420	508230	10065	2114
16_1609	26963262	577482	568842	10091	1955
16_1610	19976496	1251440	1201048	10637	4801
